# Short Term Results of Fibrin Gel Obtained from Cord Blood Units: A Preliminary in Vitro Study

**DOI:** 10.3390/bioengineering6030066

**Published:** 2019-08-02

**Authors:** Panagiotis Mallis, Ioanna Gontika, Zetta Dimou, Effrosyni Panagouli, Jerome Zoidakis, Manousos Makridakis, Antonia Vlahou, Eleni Georgiou, Vasiliki Gkioka, Catherine Stavropoulos-Giokas, Efstathios Michalopoulos

**Affiliations:** 1 Hellenic Cord Blood Bank, Biomedical Research Foundation Academy of Athens, 4 Soranou Ephessiou Street, 115 27 Athens, Greece; 2 Biotechnology division, Biomedical Research Foundation Academy of Athens, 4 Soranou Ephessiou Street, 115 27 Athens, Greece

**Keywords:** fibrin gel, platelet rich plasma, cord blood units, platelets, TGF-β1, proteomic analysis

## Abstract

Background: Recent findings have shown that the fibrin gel derived from cord blood units (CBUs) play a significant role in wound healing and tissue regeneration. The aim of this study was to standardize the fibrin gel production process in order to allow for its regular use. Methods: CBUs (*n* = 200) were assigned to 4 groups according to their initial volume. Then, a two-stage centrifugation protocol was applied in order to obtain platelet rich plasma (PRP). The concentration of platelets (PLTs), white blood cells (WBCs) and red blood cells (RBCs) were determined prior to and after the production process. In addition, targeted proteomic analysis using multiple reaction monitoring was performed. Finally, an appropriate volume of calcium gluconate was used in PRP for the production of fibrin gel. Results: The results of this study showed that high volume CBUs were characterized by greater recovery rates, concentration and number of PLTs compared to the low volume CBUs. Proteomic analysis revealed the presence of key proteins for regenerative medicine. Fibrin gel was successfully produced from CBUs of all groups. Conclusion: In this study, low volume CBUs could be an alternative source for the production of fibrin gel, which can be used in multiple regenerative medicine approaches.

## 1. Introduction

Fibrin gel, a platelet rich plasma (PRP) derivative, is mostly obtained from adult peripheral blood (APB) after a single blood collection [1,2,3]. Recently, cord blood has been proposed as an alternative source for the production of fibrin gel [4,5,6]. Fibrin gel constitutes a natural biomaterial that can be applied in various regenerative medicine approaches, including skin, cartilage and bone regeneration, wound healing and drug delivery [7,8,9]. This biomaterial can be fabricated with different degrees of stiffness, viscosity and degradation rate through the concentration adjustment of key elements such as calcium gluconate, sodium chloride (NaCl) and batroxobin [1] Recently, it has been shown that fibrin gel’s stiffness was dependent on divalent cations of Ca and Mg, and on NaCl concentration [1].

The beneficial properties of fibrin gel in regenerative medicine are mostly attributed to the increased release of several growth factors—including transforming growth factor-β1 (TGF-β1), fibroblast growth factor (FGF), platelet derived growth factor A and B (PDGF AB and BB), vascular endothelial growth factor-A (VEGF-A) and hepatocyte growth factor (HGF)—through activation of platelets (PLTs) [9,10,11]. Specifically, these growth factors are contained in α-granules of PLTs, which are secreted during the blood coagulation cascade [12]. In this context, the entrapped PLTs in the platelet plug at the site of injury are activated by the FGF and PDGF produced from the injured cells [7,12,13]. The content of α-granules are then released, thus contributing even more in wound healing [7,12,13].

Fibrin formation is initiated when thrombin, an enzyme that is abundant in plasma, cleaves fibrinogen [7,14,15,16]. Fibrinogen (Fb) is a 340 kDa plasma glycoprotein which consists of three pairs of polypeptide chains (AαBβγ)_2_, and two types of fibrinopeptides A (FpA) and B (FpB), connected with 29 disulphide bonds to the Fb central region [7,14,15,16]. During the clotting cascade, thrombin cleaves the FpA and FpB between Arg–Gly residues [14,15,16]. This in turn exposes the knobs A and B of the central region, enabling the connection of fibrin monomers [7,14,15,16]. Further association of fibrin monomers results in the development of a three-dimensional network know as fibrin. This fibrin gel is a viscoelastic scaffold, and its properties are dependent on clot structure, number of entrapped PLTs and concentration of released growth factors [14,15,16].

Fibrin gel can be produced efficiently from cord blood units that do not meet the acceptable criteria of cord blood banks (CBBs) for processing and cryostorage [6,11,17]. It is estimated that over 80% of the received cord blood units (CBUs) are characterized by low number of total nucleated cells (TNCs <120 × 10^7^ cells) and CD34 cells (<4 × 10^7^ cells), low volume (<120 mL) and low viability (<85%), thus cannot be further processed [11,18]. These discarded CBUs could serve as a source for fibrin gel production under good manufacturing practices (GMP) conditions. Furthermore, CBUs contain the same amount of PLTs (150–400 × 10^3^ PLTs/μL) as the adult peripheral blood (APB) samples, and PLTs from both have been characterized by the same proteomic profile [7,11,17]. Only some minor changes have been reported in PLTs derived from CBUs, regarding LYN, MAP3K5, and FAM129A signaling pathways, but without having any significant impact on their function [19]. 

A number of CBBs have focused on the production of fibrin gel, in order to use it as a tool for regenerative medicine approaches, worldwide [6,11,17]. However, the fibrin gel production varied between different research groups, and their results are still under debate [3,4,6]. Proper PRP isolation and fibrin gel production can be performed from CBUs with volume up to 82 mL. This is mostly attributed by the limitations of the PLTs isolation procedure. Specifically, the fibrin gel must contain high PLT number, while the red blood cell (RBC) content must be low [3,4,6]. In low volume CBUs, the PLTs cannot be isolated without having no RBC contamination, resulting to the rejection of this product. Under those circumstances, the CBUs that are used from the CBBs for fibrin gel production are restricted only to those with volume up to 82 mL and a low content of TNCs. In addition, high volume CBUs are characterized by a greater number of PLTs, thus could be the most valuable source for fibrin gel production. Although, high volume CBUs are characterized by great number of TNCs, and are more valuable being used as hematopoietic stem cell transplants, rather than as a source for fibrin gel development [6]. Based on the above data, and knowing that more than 80% of received CBUs are characterized by low volume and TNC number, it would be worthwhile to use them as a source for the fibrin gel production. For this purpose, the aim of this study was the standardization of the procedure for fibrin gel development, utilizing the low volume CBUs.

## 2. Methods

### 2.1. Cord Blood Units Collection

CBUs were collected at full-term gestation (37–40 weeks), after parental informed consent, in a collection bag (Macopharma, United Kingdom) containing 30 mL of citrate-phosphate-dextrose (CPD), and immediately distributed to Hellenic Cord Blood Bank (HCBB). The collections were performed in accordance with the ethical standards of the Greek National Ethical Committee, were approved by our Institution’s ethical board (Referene No. 1508, 5/9/2018) and were in accordance with the declaration of Helsinki. After storage at 4 °C, the units were processed within 24 h from collection. Only the CBUs that did not meet the criteria outlined by HCBB (Table S1), were used for the preparation of the PRP. 

### 2.2. Preparation of Platelet Rich Plasma and Fibrin Gel

In this study, *n* = 200, CBUs were distributed into four groups (*n* = 40 for each group) according to their initial net volume (including the anticoagulant). Group A, <81 mL; group B, 82–110 mL; group C, 111–148 mL; and group D involved CBUs that were pooled according to their blood group at a final volume of 111–148 mL. Specifically, in group D, 2 compatible CBUs were combined in order to produce 1 pooled CBU. The total number of CBUs that were combined in group D was 80, resulting in 40 pooled CBUs. 

Automated cell counting was performed in all CBUs of each group with a hematological analyzer (Abacus 5 part 380 series, Beckman Coulter, Atlanta, GA, USA) for the determination of the total number and concentration of white blood cells (WBCs), RBCs and PLTs. CBUs with a PLT concentration of less than 150 × 10^3^/μL, were excluded from the study [6]. 

Then, the CBUs were centrifuged at 210 *g* for 15 min, at room temperature (RT), and the top plasma fraction was collected and transferred in a secondary processing bar. A sample from the plasma fraction was taken for PLT counting at the hematological analyzer (Abacus 5 part 380, Beckman Coulter, Atlanta, GA, USA). Then, the plasma fraction was centrifuged at 2600 *g* for 15 min, at RT, and the supernatant platelet-poor plasma (PPP) was collected, in excess of the final target volume of the PRP. For the determination of the final volume of the PRP, the following equations were used, as previously described, with some modifications [6]:

Lower limit of PRP volume = PLT concentration in plasma fraction/(800 × 10^3^/μL)

Upper limit of PRP volume = PLT concentration in plasma fraction/(1400 × 10^3^/μL)

Target PRP volume (mL) = (Upper limit + lower limit)/2

where, the factors (800 × 10^3^/μL) and (1400 × 10^3^/μL) corresponded to the minimum and maximum PLT concentration in PRP products. Specifically, the PLT concentration in the final product should be more than 800 × 10^3^ PLTs/μL and not exceed 1400 × 10^3^ PLTs/μL, otherwise the PRP units must be discarded.

Finally, the fibrin gel was obtained after the activation of PRP samples with the addition of 10% calcium gluconate, in ratio 3:1 at 37 °C, as has been described in literature [7,17].

### 2.3. Protein Determination Using Multiple Reaction Monitoring

In order to verify if significant alterations were presented in growth factor and chemokine content between PRP derived from CBUs and APB, liquid chromatography/multiple reaction monitoring (LC/MRM) analysis was applied. For this purpose, PRP products (*n* = 8) derived from all CBU groups (A–D) were used. APB PRP samples (*n* = 8) were obtained from Evagelismos Hospital. Expired APB units were used for the production of PRP. APB collection was performed from healthy donors, following the Greek regulatory procedures for blood donation.

Initially, total protein content of PRP derived either from CBUs (*n* = 4) or APB (*n* = 4) was quantified with Bradford assays. Then, the samples were diluted with urea buffer (8 M urea, 50 mM NH_4_HCO_3_, Sigma-Aldrich, Darmstadt, Germany) to reach final volume of 20 μL. Reduction using 10 mM dithioerythritol (DTE, Sigma-Aldrich, Darmstadt, Germany) and alkylation with 40 mM iodoacetmide (Sigma-Aldrich, Darmstadt, Germany) was performed, followed by dilution with 50 mM NH_4_HCO_3_ (Sigma-Aldrich, Darmstadt, Germany) until reaching a final volume of 90 μL. Overnight trypsinization was performed in each sample with enzyme-protein ratio of 1:100. The next day, 0.1% formic acid (Sigma-Aldrich, Darmstadt, Germany) was added. Finally, the samples were desalted by zip-tip and vacuum dried (SpeedVac vacuum concentrators, Thermo Fisher Scientific, Waltham, MA, USA). 

The samples were reconstituted to a final protein concentration of 0.5 μg/mL and analyzed by LC/MRM. A total number of 31 proteins were tested for with this approach (Table S2). The protein determination was performed according to previous data in the literature with some modifications [11]. 

Skyline software and Peptideatlas repository were used for the identification of the proteotypic peptides of PRP samples. All chromatograms obtained from LC-MRM were visually inspected. The sum of peak areas of two to four transitions per peptide was used to calculate the signal intensity for the selected growth factors. A detailed list of MRM transitions is provided as supplementary material (Table S4). 

Further analysis involved the classification of the identified proteins using the Panther classification system (www.pantherdb.org).

### 2.4. Liquid Chromatography/Multiple Reaction Monitoring Setup

Agilent 1200 series (Agilent Technologies, Inc., Santa Clara, CA, USA) coupled with CS18 nano-column (150 mm × 75 μm), particle size 5 μm) was used for LC. Peptide separation and elution was performed using a 40 min 5–45% ACN/water 0.1% FA gradient at a flow rate of 300 nL/min. Six microliters of each sample (corresponding to 3 µg of total protein content) were injected. AB/MDS Sciex 4000 QTRAP coupled with a nanoelectrospray ionization source was used for the tryptic peptide analysis. The mass spectrometer was operated in MRM mode, with the first (Q1) and third quadrupole (Q3) at 0.7 unit mass resolution. Two to four transitions of each peptide were recorded. Optimum collision energies for each transition were automatically calculated by the Skyfline software.

### 2.5. Contamination Validation Study

All CBUs, APB samples and produced PRP samples were tested for HIV, HBV, HGV, HTLV-I/II, CMV, HCV, HAV, WNV, T Pallidum, syphilis, aerobic anaerobic bacteria. Furthermore, PRP samples derived from both sources were evaluated for Mycoplasma contamination and endotoxin level.

### 2.6. Evaluation of Fibrin Gel

The produced fibrin gels (*n* = 10) from each group were further evaluated. For this purpose, the time needed for the development of fibrin gel, after the addition of 10% calcium gluconate, was determined. In addition, the mean fibrin gel area was determined with the use of a digital caliper (Mitutoyo, Radionics, Ltd., Dublin, Ireland). Finally, the degradation time of fibrin gel was also estimated. The estimation of fibrin gel stability time was performed at room temperature (RT, 21–25 °C) and 37 °C. The fibrin gel stability time corresponded to hours (h). At RT, the fibrin gels from all groups were placed in open Petri dishes (Sigma-Aldrich, Darmstadt, Germany), until complete gel degradation. For the second measurements, the fibrin gels were placed in 50 mL falcon tubes (BD falcon tubes, Corning, NY, USA), and immersed into a water bath (WNB 10, Memmert, Gmbh, Schwabach, Germany) at 37 °C, until complete gel degradation. 

### 2.7. Statistical Analysis

Statistical analysis was performed using GraphPad Prism v6 (GraphPaD Software, San Diego, CA, USA). Comparisons of PRP data were performed with the unpaired Kruskal Wallis test. Statistically significant differences were obtained when the *p* value was less than 0.05. Data were presented as medians and mean ± standard deviation (SD). Pie charts of protein classifications were produced with Microsoft Excel 2016 (Microsoft Office for Windows 2016, Redmond, Washington, DC, USA). 

## 3. Results

### 3.1. Platelet Rich Plasma Data Analysis

A total number of 200 CBUs that did not fulfill the minimum criteria for processing and banking of the HCBB (Table S1) were used for the evaluation of fibrin gel production. The collection of CBUs was started in October of 2018 and ended in April of 2019. The CBUs were divided into 4 groups according to their volume (including the anticoagulant). The characteristics of each group prior and PRP production are represented in Table 1. The initial cord blood volumes in groups A to D, were 73 ± 6 mL, 97 ± 9 mL, 127 ± 11 mL and 130 ± 12 mL, respectively (Table 1). 

**Figure 1:**
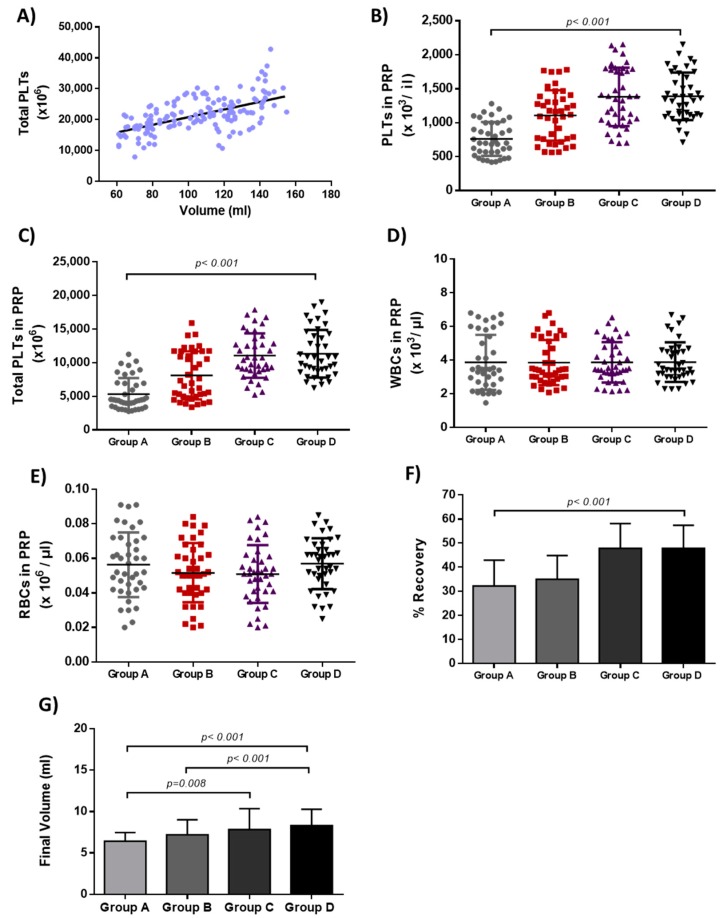
Characteristics of PRP samples from all groups. (**A**) Correlation between initial PLT number and initial cord blood (CB) volume, R^2^ = 0.3362. (**B**) PLT concentration in PRP samples derived from all groups. Statistically significant differences were observed in PLT concentration between groups A–D (*p* < 0.001). (**C**) Total number of PLTs in PRP samples derived from all groups. Statistically significant differences were observed in total PLT number between groups A–D (*p* < 0.001). (**D**) White blood cell (WBC) concentration in PRP samples derived from all groups. (**E**) Red blood cell (RBC) concentration in PRP samples derived from all groups. (**F**) PLT recovery in PRP samples. Statistically significant differences were observed in PLT recovery between all groups (*p* < 0.001). (**G**) Final volume of PRP samples. Statistically significant differences were observed between group A and group C (*p* = 0.008) and D (*p* < 0.001), and between group B and group D (*p* < 0.001).

The mean PLT concentration and the initial PLT number of all groups are represented in Table 1. Moreover, the initial PLT number was correlated positively with the initial CBU volume, as shown in Figure 1A.

After the centrifugation steps, the final volume of PRP in groups A to D was 6 ± 1 mL, 7 ± 2 mL, 8 ± 2 mL and 8 ± 2 mL, respectively (Figure 1). The median PLT number in PRP of groups A to D, was 17.3 × 10^9^, 21.5 × 10^9^, 22.6 × 10^9^ and 22.7 × 10^9^ respectively (Table 1, Figure 1). The mean of WBCs and RBCs in PRP of all groups was less than 4 × 10^3^ /μL and 1 × 10^6^/μL, respectively (Figure 1, Table S3). Accordingly, the median recovery of PLTs in PRP groups A to D, was 30%, 34%, 48% and 48%, respectively (Table 2). Statistically significant differences resulted regarding the concentration (*p* < 0.001) and number (*p* < 0.001) of PLTs, as well as volume (*p* < 0.001) and recovery (*p* < 0.001) after the PRP production, between all groups. 

### 3.2. Protein Identification

LC/MRM technology was used for the identification of proteins in PRP from CBUs and APB. A number of 25 proteins, which corresponded to 81% of the proposed proteins, were successfully identified both in PRP from CBUs, and APB samples (Table 3 and Table S4). The correspondingly identified peptide ions of each proteins are represented in Table S4. Identical proteins were identified in PRP and APB samples. 

**Figure 2:**
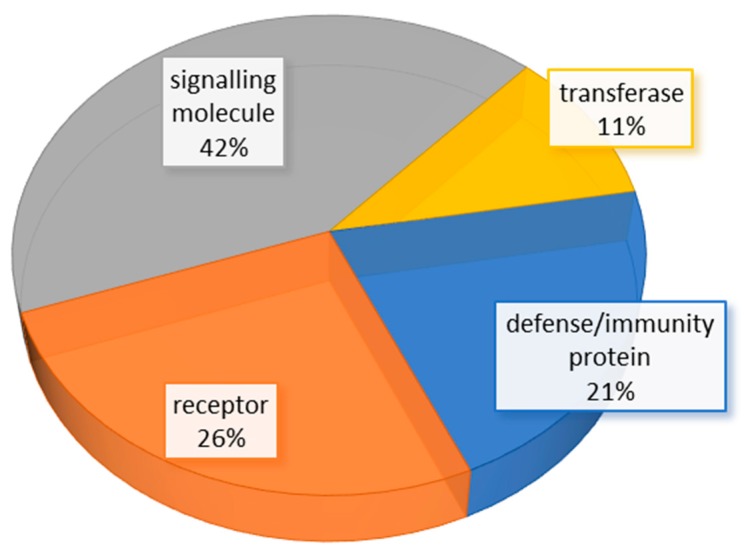
Classification of identified proteins based on the Panther classification system.

Furthermore, the identified proteins were classified using the Panther classification system. Based on their biological function, 11% of proteins were transferases, 21%, immunity/defense proteins, 26% receptors and 42% signaling molecules (Figure 2 and Table S5).

### 3.3. Platelet Rich Plasma and Adult Peripheral Blood Validation Tests

All CBUs, APB and PRP samples were negative for bacterial or viral contamination. All samples were negative for aerobic or anaerobic bacteria according to the BacT/Alert system, which were further confirmed by blood and Sabouraud agar (Table S6). In addition, PRP and APB samples were negative for HIV I/II, HBV, HGV, HTLV-I/II, CMV, HCV, HAV, WNV, and for *Treponema pallidum* and *Trypanosoma cruzi* (Table S7). The endotoxin level in PRP samples derived from both sources, was less than 2.5 EU/mL, while no mycoplasma contamination was detected (Table S7).

### 3.4. Evaluation of Fibrin Gel Production

**Figure 3:**
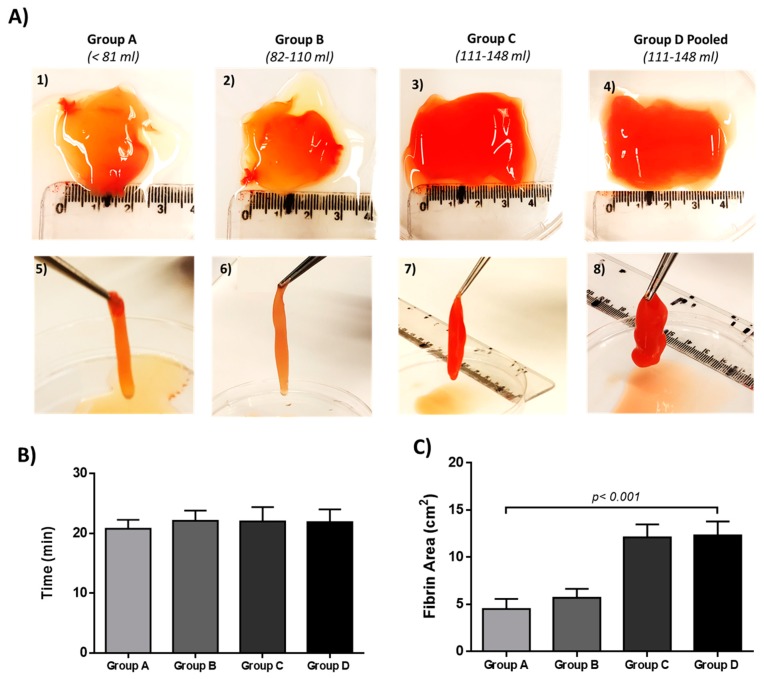
Characteristics of fibrin gel development. (**A**) Fibrin gel obtained from group A (A1, A5), group B (A2, A6), group C (A3, A7) and group D (A4, A8). (**B**) Time needed for the development of fibrin gel derived from all groups. (**C**) Fibrin gel area obtained from all groups. Statistically significant differences were observed in fibrin gel area between all groups (*p* < 0.001).

The PRP from all groups (A to D) were solidified successfully after the addition of calcium gluconate (Figure 3). The developed fibrin was characterized by a gelatinous form, which was lifted without breaking from the petri dish (Figure 3). 

The mean time needed for the fibrin development of groups A to D, was 21 ± 1 min, 22 ± 2 min, 22 ± 2 min and 22 ± 1 min, respectively (Figure 3). Furthermore, the mean covered surface of each fibrin gel was estimated. Specifically, the fibrin gel surface of groups A to D, was 5 ± 1 cm^2^, 6 ± 1 cm^2^, 12 ± 1 cm^2^ and 12 ± 1 cm^2^, respectively (Figure 3). Statistically significant differences were observed only in fibrin gel produced surface between groups A and B, with groups C (*p* < 0.001) and D (*p* < 0.001). Further evaluation involved the determination of fibrin gel stability time at RT and 37 °C. The mean degradation time of fibrin gels at RT in groups A–D was 1.3 ± 0.2 h, 2 ± 0.3 h, 4 ± 0.4 h and 4 ± 0.3 h, respectively (Table 4). The mean degradation time of fibrin gels at 37 °C in groups A–D, was 3 ± 0.3 h, 4 ± 0.4 h, 7 ± 0.4 h and 7 ± 0.4 h, respectively (Table 4). Statistically significant differences in fibrin gel degradation time between groups A–D, at RT (*p* < 0.001) and 37 °C (*p* < 0.001), were found.

## 4. Discussion

Fibrin gel has been used widely as a tool for tissue engineering and regenerative medicine approaches. At this point, fibrin gel and PRP products derived either from autologous or allogeneic sources have been used for the treatment of skin ulcers, bone and cartilage repair, and even more prominently in cosmetics surgeries [7]. Although these autologous medicinal products are considered microbiologically and virally safe, a number of practical limitations may hamper their clinical use [7,17]. In cases where repeated blood collections must be performed from patients, including the elderly, neonates and patients with cardiovascular diseases or hematological malignancies, the autologous products may be clinically inappropriate. 

Due to these drawbacks, the allogeneic fibrin gel may be used in these categories of patients. Allogeneic fibrin gel mostly is produced from expired blood units or PLT units after platelet apheresis [20,21]. However, expired blood units may increase the risk of contamination, thus the products may be characterized by low clinical effect. On the other hand, discarded CBUs from public CBBs could represent a significant source for production of fibrin gel [6,18]. It is estimated that over of 80% of the delivered CBUs in CBBs are discarded due to their low stem cell content and collected volume, thus are not appropriate for a hematopoietic stem cell transplantation.

The aim of this study was the standardization of fibrin gel production from discarded CBUs, in order to develop a highly reproducible method, irrespective of their initial volumes. Thus, low volume CBUs could be combined and used for fibrin gel production. For this purpose, a number of 200 CBUs that were delivered to HCBB, and did not meet the minimum criteria for processing and banking, were used. 

The results of this study showed that final PLT recovery is dependent on CBU volume and initial PLT number. High volume CBUs of group C and D were characterized by better initial PLT number, recovery rates and final PLT yields, when compared to low volume CBUs. Although low volume CBUs represent the majority of the received CBUs at the HCBB, these units were difficult to handle in the laboratory setting. Due to the low volume, there was a risk in obtaining high yields of WBCs and RBCs in the final PRP product. For this reason, the extraction process in low volume CBUs was not performed appropriately, thus resulting to lower recovery rates and final PLT concentration compared to the high volume CBUs. In our study, the PLT concentration of low volume CBUs (<800 × 10^6^ PLTs/μL) in the final product, was below the proposed criteria for cord blood PRP production [6], affecting in this way, its potential therapeutic use. In order to avoid the destruction of a high number of low volume units, pooling of CBUs with the same blood group was performed. 

Pooled CBUs (group D) were characterized by similar results regarding the recovery rates, initial and final PLT concentration, and total number as group C. Furthermore, all groups were characterized by no statistically significant differences in concentration of WBCs and RBCs in the final product. Then, we tried to compare our results with a multicenter clinical grade study that was accomplished by Rebulla et al [6]. In this study, CBUs with volume of 97.6 ± 20 mL were used, and resulted in PLT recovery over of 47%. However, in our study, this recovery rate was achieved only from high volume CBUs (>111 mL). This minor discrepancy in the results between these two studies, might be explained by the use of slightly different procedures. In addition, that difference may disappear if we include more CBUs in our validation study. In the study of Rebulla et al. [6], a number of 1080 CBUs were used, whereas in our study, only 200 CBUs in total were included. Moreover, both studies showed that proper PRP production could not be performed from low volume CBUs (<97 mL). All PRP samples that were produced with the proposed protocol were negative for HIV I/II, HBV, HGV, HTLV-I/II, CMV, HCV, HAV, WNV, and for *T. pallidum* and *T. cruzi*. Specifically, the endotoxin level in PRP samples was less than 2.5 EU/mL.

The next step of this study, involved the proteomic analysis of PRP products. In order to verify that PRP derived from CBUs was not different in protein content from PRP of other sources, LC/MRM technology was applied. Proteomic analysis was performed in CB PRP products and compared with the proteomic profile of PRP derived from APB. In this way, 25 proteins of the 31 initially assessed, were successfully identified both in APB and CB PRP products, indicating that both products were similar in their protein content. The identified proteins were classified as transferases (11%), defense/immunity proteins (21%), receptors (26%) and signaling molecules (42%). No discrepancy in protein identification was observed between APB and CB fibrin gel. This fact is important, regarding the therapeutic potential of fibrin gel. In the study of Stokhuijen et al. [22], differences between PLTs from APB and CBUs were observed only for intracellular proteins which belonged to metabolic pathways, and were not adhesive integrins or glycoproteins. This in turn indicated that either CB or APB PLT derived products can act in the same way, without any significant alteration to their therapeutic result. Indeed, Tadini et al. [23], Janmey et al. [7] and Perseghin et al. [24] have confirmed the beneficial effect of CB derived fibrin gel and PRP in the treatment of skin lesions produced by dystrophic epidermolysis bullosa and chronic wounds. Furthermore, the therapeutic properties of fibrin gel may be useful in bone and muscle tissue regeneration. Fibrin gel can be combined efficiently with acellular matrices. Indeed, Aulino et al. described a novel approach for muscle and bone regeneration through the use of decellularized tibialis anterior [25]. In this context, and knowing that specific growth factors, such as TGF-β1, FGF, VEGF, PDGF, and cytokines, can guide better the migration and differentiation of stem cells, the use of fibrin gel might be a beneficial approach [25]. Fibrin gel contains significant amounts of growth factors and cytokines, with defined viscoelastic properties, thus it can be used separately or in combination with various matrices in order to be used as a tool for regenerative medicine applications.

Based on their biological function, these proteins play significant role in tissue regeneration. Among them, growth factors including TGF-β1, FGF, VEGF-A, PDGF-A have been reported in literature for their potential contribution in signaling pathways responsible for cell activation, proliferation and differentiation. Moreover, that growth factor repertoire has been implicated in regulation of phosphatase and tensin homolog (PTEN), a significant signaling protein which both reduces the hypertrophic scar tissue and promotes fibroblast transdifferentiation [25]. Furthermore, the growth factors can promote, via MAP/ERK and Wnt singalling pathways, the regeneration of osteocytes and chondrocytes at the site of the injury [26]. In addition, the identified cytokine receptors and adhesion molecules can potentially contribute even more in cell proliferation and regeneration of damaged tissues [11]. 

In the current study, no matrix metaloproitenases (MMPs) were able to be identified in all PRP samples using LC/MRM. MMPs are enzymes responsible for the destruction of extracellular matrix components during the fibrosis process. These enzymes are also used by cancer cells in order to perform tumor metastasis [27]. The presence of MMPs in the PRP product could act negatively in the tissue regeneration process. The fact that no MMPs were identified in PRP from both sources, supports further its beneficial effect in tissue regeneration and wound healing. Up to now, the presence of MMPs in the PRP is still under debate. Several studies are in accordance with our findings [4,28], but evidence from other research groups confirms the presence of MMPs in PRP samples [29,30]. This phenomenon might be explained due to the different preparation protocols of PRP that are used by the research groups, worldwide. More work needs to be performed in this field in order to obtain safer results.

Finally, PRP samples from all groups of this study were solidified successfully, thus producing the fibrin gel. In less than 30 min at 37 °C, the fibrin gel was produced with the addition of appropriate volume of calcium gluconate. In addition, high volume CBUs of group C and D were able to produce wider surface gel area compared to the low volume units of group A and B. In this way, a single fibrin a gel could be administrated in a wound area of 5–12 cm^2^, while a greater number of fibrin gels could be used to wider lesions. No significant alterations in fibrin gel production were reported in the literature when thrombin or batroxobin instead of calcium glugonate, have been used [4]. In addition, fibrin gels derived from group C and D, needed greater time to be degraded when compared to those of group A and B. Owing to that, the fibrin gel content, including the growth factors and the cytokines, could be present for longer time at the site of injury, providing greater regenerative potential.

The above data indicated that CBUs with initial volumes greater than 82 mL could be used, potentially, for fibrin gel production. However, the efficacy of fibrin gel production may be significantly improved when pooled CBUs are used. Before pooling, the initial PLT concentration in CBUs must be determined. Only CBUs with concentration greater than 150 × 10^3^ PLTs/μL, can be pooled. Therefore, it is estimated that the concentration in fibrin gel will be greater than 800 × 10^3^ PLTs/μL, which has been described as a significant therapeutic dose for regenerative medicine approaches. Ideally, the PRP after its production from CBUs can be stored at −80 °C. On demand, the PRP could be thawed in a water bath at 37 °C, followed by the addition of calcium gluconate, in order to form the fibrin gel. Under that model, a PRP storage bank could be developed, where the produced fibrin gel, with the same blood group with the patients, could be administrated for regenerative medicine applications, such as wound healing, extended skin lesions, and bone and muscle regeneration. Regarding the determination of skin lesions or the establishment of bone and muscle damage, bioimpedance detection could be applied by the physicians. Bioimpedance analysis is a powerful diagnostic tool than can be used for the classification of skin lesions and mucosa damage [31]. Recently, in the study of Tatullo et al. [31], the bioimpedance detection system was used in oral lichen planus lesions that can potentially lead to malignant transformations. In that context, and by determining accurately the tissue damage in patients, fibrin gel could be administrated in different ways in order to achieve the best outcome.

Fibrin gel could be a useful tool for tissue regeneration, which can be produced with a different amount of stiffness, viscoelastic properties and degradation rates [1]. They can be achieved by modifying the key elements of solidification process, such as the concentration of calcium gluconate, batroxobin, sodium chloride, or the divalent cations of Ca and Mg [1]. In this context, the gel which will be used in various skin lesions, may be characterized by high bioabsorption rate. On the other hand, in bone tissue engineering, fibrin gels with increased stiffness would be applied. Furthermore, when fibrin gel could be used as a cell carrier, it must be characterized by a specific pore size, providing a suitable cellular microenvironment. In order to achieve that, fibrin gel could be combined with various chemical compounds, such as nanosilicates. Kerativitayanan et al. [32] presented a possible production of osteoinductive and elastomeric scaffolds with specific pore sizes by combining polyglycerol sebacate and nanoslicates.

Future experiments will include a greater number of CBUs, in order to obtain more valid conclusions regarding the process reproducibility

## 5. Conclusions

Fibrin gel is naturally derived hydrogel that can be developed in a few minutes and is characterized by unique viscoelastic properties. Its production cost is relatively low, as has been indicated in literature [6]. The potential use of low volume, discarded CBUs for fibrin gel production is of major importance. Worldwide, public CBBs accredited by the Foundation for the Accreditation of Cellular Therapy (FACT) are economically burdened by the great number of low volume CBUs that must be discarded. The results of our study indicate that the low volume CBUs can be used for fibrin gel development, which could be used as a tool by physicians in regenerative medicine approaches. With that in mind, a PRP storage bank can be established, where the PRP samples can be stored over a long time period, and thawed on demand, in order to form the fibrin gel. The produced fibrin gel could be a useful tool for physicians in various regenerative medicine approaches.

## Figures and Tables

**Table 1:** Characteristics of cord blood units prior processing, distributed in four groups. Statistically significant differences were observed in initial platelet (PLT) number between all groups (*p* < 0.05). ^a^ The values represented are medians. ^b^ The values are represented as mean ± sd.

*n* = 40/Group	Group A (<81 mL)	Group B (82–110 mL)	Group C (111–148 mL)	Group D-Pooled (111–148 mL)	*p*-Value
Initial Net Weight (g)	(78) ^a^ 77 ± 7 ^b^	(103) 103 ± 3	(131) 134 ± 12	(138) 138 ± 11	-
Initial Net Volume (mL)	(74) 73 ± 6	(97) 97 ± 9	(124) 127 ± 11	(128) 130 ± 12	-
PLTs (×10^3^/μL)	(213) 214 ± 43	(223) 223 ± 35	(187) 182 ± 55	(171) 184 ± 39	ns
Initial PLTs count (×10^6^)	(17298) 16831 ± 3601	(21510) 22687 ± 3791	(22580) 23100 ± 3659	(22672) 23850 ± 3657	<0.05

**Table 2:** Characteristics of platelet rich plasma (PRP) distributed in the four groups. Statistically significant differences were observed in final volume (*p* < 0.001), PLT concentration (*p* < 0.001), PLT number (*p* < 0.001) and recovery (*p* < 0.001) between all groups. ^a^ The values represented are medians. ^b^ The values are represented as mean ± sd.

*n* = 40/Group	Group A (<81 mL)	Group B (82–110 mL)	Group C (111–148 mL)	Group D-Pooled (111–148 mL)	*p*-Value
PRP Volume (mL)	(6) ^a^ 6 ± 1 ^b^	(7) 7 ± 2	(8) 8 ± 2	(8) 8 ± 2	<0.001
Final PLTs (×10^3^/μL)	(702) 761 ± 246	(1142) 1107 ± 367	(1366) 1382 ± 425	(1352) 1388 ± 345	<0.001
Final PLTs count (×10^6^)	(4407) 5331 ± 2355	(7151) 8120 ± 3528	(10568) 11066 ± 3260	(10523) 11345 ± 3496	<0.001
Recovery (%)	(30) 32 ± 11	(34) 35 ± 10	(48) 48 ± 10	(48) 48 ± 11	<0.001

**Table 3:** List of protein identifications in adult peripheral blood (APB) and cord blood (CB) PRP.

No.	Growth Factors	Entry Name	Ascession Number
1	Tumor Neccrosis Factor A (TNF A)	TNFA_HUMAN	P01375
2	Interleukin-1A (IL-1A)	IL1A_HUMAN	P01583
3	Interleukin-1B (IL-1B)	IL1B_HUMAN	P01584
4	Interleukin-2 (IL-2)	IL2_HUMAN	P60568
5	Interleukin-6 (IL-6)	IL6_HUMAN	P05231
7	Interleukin-8 (IL-8)	IL8_HUMAN	P10145
9	Tumour necrosis factor receptor type 1-associated DEATH domain protein (TRADD )	TRADD_HUMAN	Q15628
10	Interleukin-1 Receptor (IL-1R)	IL1R1_HUMAN	P14778
11	Interleukin-2 Receptor (IL-2GR)	IL2RG_HUMAN	P31785
12	Interleukin-6 Receptor (IL-6R)	IL6RA_HUMAN	P08887
15	Interleukin-10 Receptor 1 (IL-10R1)	I10R1_HUMAN	Q13651
16	Interleukin-10 Receptor 2 (IL-10R2)	I10R2_HUMAN	Q08334
17	Vascular Endothelial Growth Factor A (VEGF-A)	VEGFA_HUMAN	P15692
18	Vascular Cell Adhesion protein 1 precursor (VCAM-1 )	VCAM1_HUMAN	P19320
19	Intracellular Cell Adhesion protein 1 precursor (ICAM-1)	ICAM1_HUMAN	P05362
20	Platelet Derived Growth Factor AA (PDGF-AA)	PDGFA_HUMAN	P04085
21	Transforming Growth Factor B1 (TGF-B1)	TGFB1_HUMAN	P01137
22	Fibroblast Growth Factor 2 (FGF2)	FGF2_HUMAN	P09038
23	C-C motif chemokine receptor 1 (CCR1)	CCR1_HUMAN	P32246
24	Transforming Growth Factor-B receptor 1 (TGF-B R1)	TGFR1_HUMAN	P36897
25	Transforming Growth Factor-B receptor 2 (TGF-B R2)	TGFR2_HUMAN	P37173

**Table 4:** Fibrin gel stability time at 25 °C and 37 °C. Statistically significant differences in fibrin gel stability time at 25 °C (*p* < 0.001) and 37 °C (*p* < 0.001) were observed between all groups.

Fibrin Gel Degradation Temperature	Group A (h)	Group B (h)	Group C (h)	Group D (h)	*p*-Value
RT (21–25 °C)	1.3 ± 0.2	2 ± 0.3	4 ± 0.4	4 ± 0.3	<0.001
37 °C	3 ± 0.3	4 ± 0.4	7 ± 0.4	7 ± 0.4	<0.001

## Supplementary Materials

The following are available online at https://www.mdpi.com/2306-5354/6/3/66/s1: Table S1: Acceptable range of results outlined by the Hellenic Cord Blood Bank for processing and storage of cord blood units. Table S2: List of proteins for identification with LC/MRM. Table S3: WBCs and RBCs prior and after processing. * The values are represented as median. † The values are represented as mean ± sd. Table S4: List of parent ions and fragments used in MRM analysis of PB-PL and CB-PL growth factors. Table S5: Protein classification based on their biological functions. Table S6: Validation test for CBUs and APB samples. Table S7: Validation test for PRP derived from CBUs and APB samples.
