# Supplementary material for: Short Term Results of Fibrin Gel Obtained from Cord Blood Units: A Preliminary in Vitro Study
Source: Bioengineering (Basel). 2019 Aug 2;6(3):66. doi: 10.3390/bioengineering6030066 (PMC6783834; doi:10.3390/bioengineering6030066)
Supplement: Supplementary file 1 [file bioengineering-06-00066-s001.pdf]

Table S1. Acceptable range of results outlined by the Hellenic Cord Blood Bank for processing and storage of cord blood units.

Criteria	Unrelated Allogeneic	Related Allogeneic
**Collection bag mixed weight**	120 – 280 gr	100 – 220 gr
**WBC absolute count**	120 – 250 x 10^7 cells	100 – 250 x 10^7 cells
**WBC concentration**	13 – 29.5 cells/μL	10 – 29.5 cells/μL
**Cell Viability**	85 – 100%	85 – 100%

Table S2. List of proteins for identification with LC/MRM.

No.	GROWTH FACTORS	ENTRY NAME	ASCESSION NUMBER
1	TNF-A	TNFA_HUMAN	P01375
2	IL-1A	IL1A_HUMAN	P01583
3	IL-1B	IL1B_HUMAN	P01584
4	IL-2	IL2_HUMAN	P60568
5	IL-6	IL6_HUMAN	P05231
6	IL-7	IL7_HUMAN	P13232
7	IL-8	IL8_HUMAN	P10145
8	IL-10	IL10_HUMAN	P22301
9	TRADD	TRADD_HUMAN	Q15628
10	IL-1R	IL1R1_HUMAN	P14778
11	IL-2GR	IL2RG_HUMAN	P31785
12	IL-6R	IL6RA_HUMAN	P08887
13	IL-7R	IL7RA_HUMAN	P16871
14	IL-8R	CXCR2_HUMAN	P25025
15	IL-10R1	I10R1_HUMAN	Q13651
16	IL-10R2	I10R2_HUMAN	Q08334
17	VEGF-A	VEGFA_HUMAN	P15692
18	SVCAM-1	VCAM1_HUMAN	P19320
19	SICAM-1	ICAM1_HUMAN	P05362
20	PDGF-AA	PDGFA_HUMAN	P04085
21	TGF-B1	TGFB1_HUMAN	P01137
22	FGF2	FGF2_HUMAN	P09038
23	CCR1	CCR1_HUMAN	P32246
24	TGF-B R2	TGFR2_HUMAN	P37173
25	TGF-B R1	TGFR1_HUMAN	P36897
26	MMP-1	MMP1_HUMAN	P03956
27	MMP-2	MMP2_HUMAN	P08253
28	MMP-3	MMP3_HUMAN	P08254
29	TIMP1	TIMP1_HUMAN	P01033
30	TIMP2	TIMP2_HUMAN	P16035
31	TIMP3	TIMP3_HUMAN	P35625

Table S3. WBCs and RBCs prior and after processing. * The values are represented as median. † The values are represented as mean ± sd.

n=40/group	**Group A** *(< 81 ml)*	**Group B** *(82-110 ml)*	**Group C** *(111-148 ml)*	**Group D-Pooled** *(111-148 ml)*	***p value***
**Initial WBCs** ($x\ 10^3$ / μl)	(7) 7 ± 1	(8) 8 ± 1	(8) 8 ± 2	(8) 8 ± 1	ns
**Initial RBCs** ($x\ 10^6$)	(3) 3 ± 1	(3) 3 ± 1	(3) 4 ± 1	(4) 4 ± 1	ns
**Final WBCs** ($x\ 10^3$ / μl)	(3) 4 ± 2	(4) 4 ± 1	(4) 4 ± 1	(4) 4 ± 1	ns
**Final RBCs** ($x\ 10^6$ / μl)	<< 1	<< 1	<< 1	<< 1	ns

Table S4. List of parent ions and fragments used in MRM analysis of PB-PL and CB-PL growth factors.

Parent Ion (m/z) (z=1)	Fragment ion (m/z)	Fragment ion information
565,296027	888,442137	sp|P09919|CSF3_HUMAN.IQGDGAALQEK.+2y9.light
565,296027	716,39373	sp|P09919|CSF3_HUMAN.IQGDGAALQEK.+2y7.light
565,296027	588,335152	sp|P09919|CSF3_HUMAN.IQGDGAALQEK.+2y5.light
565,296027	517,298038	sp|P09919|CSF3_HUMAN.IQGDGAALQEK.+2y4.light
565,296027	508,753995	sp|P09919|CSF3_HUMAN.IQGDGAALQEK.+2y10+2.light
761,896637	1031,547999	sp|P01579|IFNG_HUMAN.LTNYSVTDLNVQR.+2y9.light
761,896637	944,51597	sp|P01579|IFNG_HUMAN.LTNYSVTDLNVQR.+2y8.light
761,896637	845,447556	sp|P01579|IFNG_HUMAN.LTNYSVTDLNVQR.+2y7.light
761,896637	744,399878	sp|P01579|IFNG_HUMAN.LTNYSVTDLNVQR.+2y6.light
761,896637	516,288871	sp|P01579|IFNG_HUMAN.LTNYSVTDLNVQR.+2y4.light
607,669478	786,435595	sp|P01579|IFNG_HUMAN.AIHELIQVMAELSPAAK.+3y8.light

607,669478	715,398481	sp|P01579|IFNG_HUMAN.AIHELIQVMAELSPAAK.+3y7.light
607,669478	586,355888	sp|P01579|IFNG_HUMAN.AIHELIQVMAELSPAAK.+3y6.light
607,669478	473,271824	sp|P01579|IFNG_HUMAN.AIHELIQVMAELSPAAK.+3y5.light
607,669478	386,239795	sp|P01579|IFNG_HUMAN.AIHELIQVMAELSPAAK.+3y4.light
499,224016	781,366135	sp|P15260|INGR1_HUMAN.SEEFAVC[CAM]R.+2y6.light
499,224016	652,323542	sp|P15260|INGR1_HUMAN.SEEFAVC[CAM]R.+2y5.light
499,224016	505,255128	sp|P15260|INGR1_HUMAN.SEEFAVC[CAM]R.+2y4.light
499,224016	434,218014	sp|P15260|INGR1_HUMAN.SEEFAVC[CAM]R.+2y3.light
499,224016	335,1496	sp|P15260|INGR1_HUMAN.SEEFAVC[CAM]R.+2y2.light
887,966728	1199,59428	sp|P15260|INGR1_HUMAN.YVSLITSYQPFSLEK.+2y10.light
887,966728	1098,546602	sp|P15260|INGR1_HUMAN.YVSLITSYQPFSLEK.+2y9.light
887,966728	1011,514573	sp|P15260|INGR1_HUMAN.YVSLITSYQPFSLEK.+2y8.light
887,966728	848,451245	sp|P15260|INGR1_HUMAN.YVSLITSYQPFSLEK.+2y7.light
887,966728	720,392667	sp|P15260|INGR1_HUMAN.YVSLITSYQPFSLEK.+2y6.light
620,854043	871,499592	sp|P01375|TNFA_HUMAN.ANALLANGVELR.+2y8.light
620,854043	758,415528	sp|P01375|TNFA_HUMAN.ANALLANGVELR.+2y7.light
620,854043	687,378414	sp|P01375|TNFA_HUMAN.ANALLANGVELR.+2y6.light
620,854043	573,335487	sp|P01375|TNFA_HUMAN.ANALLANGVELR.+2y5.light
620,854043	417,245609	sp|P01375|TNFA_HUMAN.ANALLANGVELR.+2y3.light
911,977961	1210,694269	sp|P01583|IL1A_HUMAN.NYFTSVAHPNLFIATK.+2y11.light
911,977961	1111,625855	sp|P01583|IL1A_HUMAN.NYFTSVAHPNLFIATK.+2y10.light
911,977961	1040,588741	sp|P01583|IL1A_HUMAN.NYFTSVAHPNLFIATK.+2y9.light

911,977961	903,529829	sp|P01583|IL1A_HUMAN.NYFTSVAHPNLFIATK.+2y8.light
911,977961	773,424833	sp|P01583|IL1A_HUMAN.NYFTSVAHPNLFIATK.+2y14+2.light
612,320656	1023,517944	sp|P01584|IL1B_HUMAN.SLVMSGPYELK.+2y9.light
612,320656	924,44953	sp|P01584|IL1B_HUMAN.SLVMSGPYELK.+2y8.light
612,320656	793,409046	sp|P01584|IL1B_HUMAN.SLVMSGPYELK.+2y7.light
612,320656	706,377017	sp|P01584|IL1B_HUMAN.SLVMSGPYELK.+2y6.light
612,320656	512,26261	sp|P01584|IL1B_HUMAN.SLVMSGPYELK.+2y9+2.light
528,942032	787,41961	sp|P01584|IL1B_HUMAN.DDKPTLQLESVDPK.+3y7.light
528,942032	674,335546	sp|P01584|IL1B_HUMAN.DDKPTLQLESVDPK.+3y6.light
528,942032	545,292953	sp|P01584|IL1B_HUMAN.DDKPTLQLESVDPK.+3y5.light
528,942032	458,260925	sp|P01584|IL1B_HUMAN.DDKPTLQLESVDPK.+3y4.light
528,942032	677,882467	sp|P01584|IL1B_HUMAN.DDKPTLQLESVDPK.+3y12+2.light
874,126544	1130,605179	sp|P60568|IL2_HUMAN.HLQC[CAM]LEEELKPLEEVLNLAQSK.+3y10.light
874,126544	773,451579	sp|P60568|IL2_HUMAN.HLQC[CAM]LEEELKPLEEVLNLAQSK.+3y7.light
874,126544	1185,61469	sp|P60568|IL2_HUMAN.HLQC[CAM]LEEELKPLEEVLNLAQSK.+3y20+2.light
874,126544	855,985452	sp|P60568|IL2_HUMAN.HLQC[CAM]LEEELKPLEEVLNLAQSK.+3y15+2.light
874,126544	791,464155	sp|P60568|IL2_HUMAN.HLQC[CAM]LEEELKPLEEVLNLAQSK.+3y14+2.light
663,356616	1012,542185	sp|P05231|IL6_HUMAN.EALAENNLNLPK.+2y9.light
663,356616	941,505071	sp|P05231|IL6_HUMAN.EALAENNLNLPK.+2y8.light
663,356616	812,462478	sp|P05231|IL6_HUMAN.EALAENNLNLPK.+2y7.light
663,356616	471,292559	sp|P05231|IL6_HUMAN.EALAENNLNLPK.+2y4.light
663,356616	563,316763	sp|P05231|IL6_HUMAN.EALAENNLNLPK.+2y10+2.light

1107,096262	1197,626249	sp|P05231|IL6_HUMAN.IITGLLEFEVYLEYLQNR.+2y9.light
1107,096262	935,494506	sp|P05231|IL6_HUMAN.IITGLLEFEVYLEYLQNR.+2y7.light
1107,096262	822,410442	sp|P05231|IL6_HUMAN.IITGLLEFEVYLEYLQNR.+2y6.light
1107,096262	693,367849	sp|P05231|IL6_HUMAN.IITGLLEFEVYLEYLQNR.+2y5.light
1107,096262	530,304521	sp|P05231|IL6_HUMAN.IITGLLEFEVYLEYLQNR.+2y4.light
883,958911	1047,550307	sp|P10145|IL8_HUMAN.VIESGPHC[CAM]ANTEIIVK.+2y9.light
883,958911	834,424704	sp|P10145|IL8_HUMAN.VIESGPHC[CAM]ANTEIIVK.+2y15+2.light
883,958911	777,882672	sp|P10145|IL8_HUMAN.VIESGPHC[CAM]ANTEIIVK.+2y14+2.light
883,958911	713,361376	sp|P10145|IL8_HUMAN.VIESGPHC[CAM]ANTEIIVK.+2y13+2.light
883,958911	641,334629	sp|P10145|IL8_HUMAN.VIESGPHC[CAM]ANTEIIVK.+2y11+2.light
730,852861	827,436992	sp|Q15628|TRADD_HUMAN.C[CAM]LSC[CAM]ILAQQPDR.+2y7.light
730,852861	714,352928	sp|Q15628|TRADD_HUMAN.C[CAM]LSC[CAM]ILAQQPDR.+2y6.light
730,852861	643,315814	sp|Q15628|TRADD_HUMAN.C[CAM]LSC[CAM]ILAQQPDR.+2y5.light
730,852861	515,257236	sp|Q15628|TRADD_HUMAN.C[CAM]LSC[CAM]ILAQQPDR.+2y4.light
730,852861	387,198659	sp|Q15628|TRADD_HUMAN.C[CAM]LSC[CAM]ILAQQPDR.+2y3.light
733,885541	1004,552356	sp|Q15628|TRADD_HUMAN.EGLYEQAFQLLR.+2y8.light
733,885541	875,509763	sp|Q15628|TRADD_HUMAN.EGLYEQAFQLLR.+2y7.light
733,885541	747,451185	sp|Q15628|TRADD_HUMAN.EGLYEQAFQLLR.+2y6.light
733,885541	676,414071	sp|Q15628|TRADD_HUMAN.EGLYEQAFQLLR.+2y5.light
733,885541	529,345657	sp|Q15628|TRADD_HUMAN.EGLYEQAFQLLR.+2y4.light
872,458516	1195,100073	sp|P14778|IL1R1_HUMAN.IILVSSANEIDVRPC[CAM]PLNPNEHK.+3y21+2.light
872,458516	1138,558041	sp|P14778|IL1R1_HUMAN.IILVSSANEIDVRPC[CAM]PLNPNEHK.+3y20+2.light

872,458516	1089,023834	sp|P14778|IL1R1_HUMAN.IILVSSANEIDVRPC[CAM]PLNPNEHK.+3y19+2.light
872,458516	730,874984	sp|P14778|IL1R1_HUMAN.IILVSSANEIDVRPC[CAM]PLNPNEHK.+3y12+2.light
872,458516	681,340777	sp|P14778|IL1R1_HUMAN.IILVSSANEIDVRPC[CAM]PLNPNEHK.+3y11+2.light
742,335358	859,413085	sp|P14778|IL1R1_HUMAN.VEDSGHYYC[CAM]VVR.+2y6.light
742,335358	696,349757	sp|P14778|IL1R1_HUMAN.VEDSGHYYC[CAM]VVR.+2y5.light
742,335358	628,279854	sp|P14778|IL1R1_HUMAN.VEDSGHYYC[CAM]VVR.+2y10+2.light
742,335358	570,766383	sp|P14778|IL1R1_HUMAN.VEDSGHYYC[CAM]VVR.+2y9+2.light
742,335358	498,739637	sp|P14778|IL1R1_HUMAN.VEDSGHYYC[CAM]VVR.+2y7+2.light
642,274733	767,368243	sp|P31785|IL2RG_HUMAN.TDWDHSWTEQSVDYR.+3y6.light
642,274733	552,277637	sp|P31785|IL2RG_HUMAN.TDWDHSWTEQSVDYR.+3y4.light
642,274733	453,209223	sp|P31785|IL2RG_HUMAN.TDWDHSWTEQSVDYR.+3y3.light
642,274733	338,18228	sp|P31785|IL2RG_HUMAN.TDWDHSWTEQSVDYR.+3y2.light
642,274733	854,87115	sp|P31785|IL2RG_HUMAN.TDWDHSWTEQSVDYR.+3y13+2.light
972,4683	1054,542854	sp|P08887|IL6RA_HUMAN.DLQHHC[CAM]VIHDAWSGLR.+2y9.light
972,4683	941,45879	sp|P08887|IL6RA_HUMAN.DLQHHC[CAM]VIHDAWSGLR.+2y8.light
972,4683	689,372935	sp|P08887|IL6RA_HUMAN.DLQHHC[CAM]VIHDAWSGLR.+2y6.light
972,4683	914,954829	sp|P08887|IL6RA_HUMAN.DLQHHC[CAM]VIHDAWSGLR.+2y15+2.light
972,4683	794,383508	sp|P08887|IL6RA_HUMAN.DLQHHC[CAM]VIHDAWSGLR.+2y13+2.light
891,540828	856,561464	sp|Q13651|I10R1_HUMAN.MLPC[CAM]LVVLLAALLSLR.+2y8.light
891,540828	743,4774	sp|Q13651|I10R1_HUMAN.MLPC[CAM]LVVLLAALLSLR.+2y7.light
891,540828	601,403172	sp|Q13651|I10R1_HUMAN.MLPC[CAM]LVVLLAALLSLR.+2y5.light
891,540828	488,319108	sp|Q13651|I10R1_HUMAN.MLPC[CAM]LVVLLAALLSLR.+2y4.light

891,540828	584,394816	sp|Q13651|I10R1_HUMAN.MLPC[CAM]LVVLLAALLSLR.+2y11+2.light
702,369526	1176,604785	sp|Q08334|I10R2_HUMAN.NILQWESPAFAK.+2y10.light
702,369526	1063,520721	sp|Q08334|I10R2_HUMAN.NILQWESPAFAK.+2y9.light
702,369526	935,462144	sp|Q08334|I10R2_HUMAN.NILQWESPAFAK.+2y8.light
702,369526	749,382831	sp|Q08334|I10R2_HUMAN.NILQWESPAFAK.+2y7.light
702,369526	620,340238	sp|Q08334|I10R2_HUMAN.NILQWESPAFAK.+2y6.light
686,837536	1122,524821	sp|P15692|VEGFA_HUMAN.HLFVQDPQTC[CAM]K.+2y9.light
686,837536	975,456407	sp|P15692|VEGFA_HUMAN.HLFVQDPQTC[CAM]K.+2y8.light
686,837536	876,387993	sp|P15692|VEGFA_HUMAN.HLFVQDPQTC[CAM]K.+2y7.light
686,837536	748,329415	sp|P15692|VEGFA_HUMAN.HLFVQDPQTC[CAM]K.+2y6.light
686,837536	633,302472	sp|P15692|VEGFA_HUMAN.HLFVQDPQTC[CAM]K.+2y5.light
788,35903	1188,553144	sp|P10147|CCL3_HUMAN.QVC[CAM]ADPSEEWVQK.+2y10.light
788,35903	1117,51603	sp|P10147|CCL3_HUMAN.QVC[CAM]ADPSEEWVQK.+2y9.light
788,35903	1002,489087	sp|P10147|CCL3_HUMAN.QVC[CAM]ADPSEEWVQK.+2y8.light
788,35903	560,319108	sp|P10147|CCL3_HUMAN.QVC[CAM]ADPSEEWVQK.+2y4.light
788,35903	674,795534	sp|P10147|CCL3_HUMAN.QVC[CAM]ADPSEEWVQK.+2y11+2.light
894,763383	721,424302	sp|P13236|CCL4_HUMAN.NFVVDYYETSSLC[CAM]SQPAVVFQTK.+3y6.light
894,763383	622,355888	sp|P13236|CCL4_HUMAN.NFVVDYYETSSLC[CAM]SQPAVVFQTK.+3y5.light
894,763383	523,287474	sp|P13236|CCL4_HUMAN.NFVVDYYETSSLC[CAM]SQPAVVFQTK.+3y4.light
894,763383	1211,085765	sp|P13236|CCL4_HUMAN.NFVVDYYETSSLC[CAM]SQPAVVFQTK.+3y21+2.light
894,763383	1161,551558	sp|P13236|CCL4_HUMAN.NFVVDYYETSSLC[CAM]SQPAVVFQTK.+3y20+2.light
824,445628	1219,65688	sp|P19320|VCAM1_HUMAN.SLEVTFTPVIEDIGK.+2y11.light

824,445628	1118,609202	sp|P19320|VCAM1_HUMAN.SLEVTFTPVIEDIGK.+2y10.light
824,445628	971,540788	sp|P19320|VCAM1_HUMAN.SLEVTFTPVIEDIGK.+2y9.light
824,445628	870,49311	sp|P19320|VCAM1_HUMAN.SLEVTFTPVIEDIGK.+2y8.light
824,445628	659,866285	sp|P19320|VCAM1_HUMAN.SLEVTFTPVIEDIGK.+2y12+2.light
756,414242	626,362036	sp|P19320|VCAM1_HUMAN.EVELIVQEKPFTVEISPGPR.+3y6.light
756,414242	513,277972	sp|P19320|VCAM1_HUMAN.EVELIVQEKPFTVEISPGPR.+3y5.light
756,414242	426,245943	sp|P19320|VCAM1_HUMAN.EVELIVQEKPFTVEISPGPR.+3y4.light
756,414242	842,456861	sp|P19320|VCAM1_HUMAN.EVELIVQEKPFTVEISPGPR.+3y15+2.light
756,414242	600,324588	sp|P19320|VCAM1_HUMAN.EVELIVQEKPFTVEISPGPR.+3y11+2.light
706,864077	1051,520721	sp|P05362|ICAM1_HUMAN.TFLTVYWTPER.+2y8.light
706,864077	851,404629	sp|P05362|ICAM1_HUMAN.TFLTVYWTPER.+2y6.light
706,864077	688,3413	sp|P05362|ICAM1_HUMAN.TFLTVYWTPER.+2y5.light
706,864077	502,261987	sp|P05362|ICAM1_HUMAN.TFLTVYWTPER.+2y4.light
706,864077	401,214309	sp|P05362|ICAM1_HUMAN.TFLTVYWTPER.+2y3.light
924,469643	1208,699748	sp|P05362|ICAM1_HUMAN.SFSC[CAM]SATLEVAGQLIHK.+2y11.light
924,469643	994,568006	sp|P05362|ICAM1_HUMAN.SFSC[CAM]SATLEVAGQLIHK.+2y9.light
924,469643	865,525413	sp|P05362|ICAM1_HUMAN.SFSC[CAM]SATLEVAGQLIHK.+2y8.light
924,469643	766,456999	sp|P05362|ICAM1_HUMAN.SFSC[CAM]SATLEVAGQLIHK.+2y7.light
924,469643	695,419885	sp|P05362|ICAM1_HUMAN.SFSC[CAM]SATLEVAGQLIHK.+2y6.light
763,052428	1014,507714	sp|P04085|PDGFA_HUMAN.SQVDPTSANFLIWPPC[CAM]VEVK.+3y8.light
763,052428	828,428401	sp|P04085|PDGFA_HUMAN.SQVDPTSANFLIWPPC[CAM]VEVK.+3y7.light
763,052428	731,375637	sp|P04085|PDGFA_HUMAN.SQVDPTSANFLIWPPC[CAM]VEVK.+3y6.light

763,052428	507,757495	sp|P04085|PDGFA_HUMAN.SQVDPTSANFLIWPPC[CAM]VEVK.+3y8+2.light
763,052428	414,717839	sp|P04085|PDGFA_HUMAN.SQVDPTSANFLIWPPC[CAM]VEVK.+3y7+2.light
625,321522	1002,573091	sp|P13501|CCL5_HUMAN.C[CAM]SNPAVVFVTR.+2y9.light
625,321522	888,530164	sp|P13501|CCL5_HUMAN.C[CAM]SNPAVVFVTR.+2y8.light
625,321522	720,440286	sp|P13501|CCL5_HUMAN.C[CAM]SNPAVVFVTR.+2y6.light
625,321522	522,303458	sp|P13501|CCL5_HUMAN.C[CAM]SNPAVVFVTR.+2y4.light
625,321522	375,235044	sp|P13501|CCL5_HUMAN.C[CAM]SNPAVVFVTR.+2y3.light
825,907733	1207,606576	sp|P01137|TGFB1_HUMAN.DNTLQVDINGFTTGR.+2y11.light
825,907733	1079,547999	sp|P01137|TGFB1_HUMAN.DNTLQVDINGFTTGR.+2y10.light
825,907733	980,479585	sp|P01137|TGFB1_HUMAN.DNTLQVDINGFTTGR.+2y9.light
825,907733	752,368578	sp|P01137|TGFB1_HUMAN.DNTLQVDINGFTTGR.+2y7.light
825,907733	434,235772	sp|P01137|TGFB1_HUMAN.DNTLQVDINGFTTGR.+2y4.light
594,823897	960,529512	sp|P01137|TGFB1_HUMAN.VEQLSNMIVR.+2y8.light
594,823897	832,470934	sp|P01137|TGFB1_HUMAN.VEQLSNMIVR.+2y7.light
594,823897	719,38687	sp|P01137|TGFB1_HUMAN.VEQLSNMIVR.+2y6.light
594,823897	632,354842	sp|P01137|TGFB1_HUMAN.VEQLSNMIVR.+2y5.light
594,823897	480,768394	sp|P01137|TGFB1_HUMAN.VEQLSNMIVR.+2y8+2.light
637,294016	1031,454098	sp|P09038|FGF2_HUMAN.LESNNYNTYR.+2y8.light
637,294016	830,379142	sp|P09038|FGF2_HUMAN.LESNNYNTYR.+2y6.light
637,294016	716,336215	sp|P09038|FGF2_HUMAN.LESNNYNTYR.+2y5.light
637,294016	553,272886	sp|P09038|FGF2_HUMAN.LESNNYNTYR.+2y4.light
637,294016	439,229959	sp|P09038|FGF2_HUMAN.LESNNYNTYR.+2y3.light

545,820095	906,511736	sp|P09038|FGF2_HUMAN.AILFLPMSAK.+2y8.light
545,820095	793,427672	sp|P09038|FGF2_HUMAN.AILFLPMSAK.+2y7.light
545,820095	646,359259	sp|P09038|FGF2_HUMAN.AILFLPMSAK.+2y6.light
545,820095	510,301538	sp|P09038|FGF2_HUMAN.AILFLPMSAK.+2y9+2.light
545,820095	453,759506	sp|P09038|FGF2_HUMAN.AILFLPMSAK.+2y8+2.light
772,341991	1216,497286	sp|P35968|VGFR2_HUMAN.VEVTEC[CAM]SDGLFC[CAM]K.+2y10.light
772,341991	1115,449607	sp|P35968|VGFR2_HUMAN.VEVTEC[CAM]SDGLFC[CAM]K.+2y9.light
772,341991	986,407014	sp|P35968|VGFR2_HUMAN.VEVTEC[CAM]SDGLFC[CAM]K.+2y8.light
772,341991	454,211866	sp|P35968|VGFR2_HUMAN.VEVTEC[CAM]SDGLFC[CAM]K.+2y3.light
772,341991	658,286488	sp|P35968|VGFR2_HUMAN.VEVTEC[CAM]SDGLFC[CAM]K.+2y11+2.light
740,887749	1034,551687	sp|P35968|VGFR2_HUMAN.VEAFFIIEGAQEK.+2y9.light
740,887749	887,483273	sp|P35968|VGFR2_HUMAN.VEAFFIIEGAQEK.+2y8.light
740,887749	774,399209	sp|P35968|VGFR2_HUMAN.VEAFFIIEGAQEK.+2y7.light
740,887749	661,315145	sp|P35968|VGFR2_HUMAN.VEAFFIIEGAQEK.+2y6.light
740,887749	532,272552	sp|P35968|VGFR2_HUMAN.VEAFFIIEGAQEK.+2y5.light
749,896637	1213,653526	sp|P17948|VGFR1_HUMAN.LGDLLQANVQQDGK.+2y11.light
749,896637	1100,569462	sp|P17948|VGFR1_HUMAN.LGDLLQANVQQDGK.+2y10.light
749,896637	987,485398	sp|P17948|VGFR1_HUMAN.LGDLLQANVQQDGK.+2y9.light
749,896637	859,426821	sp|P17948|VGFR1_HUMAN.LGDLLQANVQQDGK.+2y8.light
749,896637	788,389707	sp|P17948|VGFR1_HUMAN.LGDLLQANVQQDGK.+2y7.light
835,164666	694,413403	sp|P32246|CCR1_HUMAN.LNLFGLVLPLLVMIIC[CAM]YTGIIK.+3y6.light
835,164666	817,474623	sp|P32246|CCR1_HUMAN.LNLFGLVLPLLVMIIC[CAM]YTGIIK.+3y14+2.light

835,164666	768,948241	sp|P32246|CCR1_HUMAN.LNLFGLVLPLLVMIIC[CAM]YTGIIK.+3y13+2.light
835,164666	712,406209	sp|P32246|CCR1_HUMAN.LNLFGLVLPLLVMIIC[CAM]YTGIIK.+3y12+2.light
835,164666	655,864177	sp|P32246|CCR1_HUMAN.LNLFGLVLPLLVMIIC[CAM]YTGIIK.+3y11+2.light
858,435595	1205,604845	sp|P37173|TGFR2_HUMAN.LPYHDFILEDAASPK.+2y11.light
858,435595	1090,577902	sp|P37173|TGFR2_HUMAN.LPYHDFILEDAASPK.+2y10.light
858,435595	830,425424	sp|P37173|TGFR2_HUMAN.LPYHDFILEDAASPK.+2y8.light
858,435595	801,893563	sp|P37173|TGFR2_HUMAN.LPYHDFILEDAASPK.+2y14+2.light
858,435595	671,835516	sp|P37173|TGFR2_HUMAN.LPYHDFILEDAASPK.+2y12+2.light
922,410414	967,466577	sp|P37173|TGFR2_HUMAN.NDLTC[CAM]C[CAM]LC[CAM]DFGLSLR.+2y8.light
922,410414	807,435929	sp|P37173|TGFR2_HUMAN.NDLTC[CAM]C[CAM]LC[CAM]DFGLSLR.+2y7.light
922,410414	692,408986	sp|P37173|TGFR2_HUMAN.NDLTC[CAM]C[CAM]LC[CAM]DFGLSLR.+2y6.light
922,410414	545,340572	sp|P37173|TGFR2_HUMAN.NDLTC[CAM]C[CAM]LC[CAM]DFGLSLR.+2y5.light
922,410414	375,235044	sp|P37173|TGFR2_HUMAN.NDLTC[CAM]C[CAM]LC[CAM]DFGLSLR.+2y3.light
1015,013457	960,485733	sp|P37173|TGFR2_HUMAN.LDPTLSVDDLANSGQVGTAR.+2y10.light
1015,013457	889,448619	sp|P37173|TGFR2_HUMAN.LDPTLSVDDLANSGQVGTAR.+2y9.light
1015,013457	775,405691	sp|P37173|TGFR2_HUMAN.LDPTLSVDDLANSGQVGTAR.+2y8.light
1015,013457	688,373663	sp|P37173|TGFR2_HUMAN.LDPTLSVDDLANSGQVGTAR.+2y7.light
1015,013457	900,957954	sp|P37173|TGFR2_HUMAN.LDPTLSVDDLANSGQVGTAR.+2y18+2.light
721,37534	1175,641899	sp|P36897|TGFR1_HUMAN.HENILGFIAADNK.+2y11.light
721,37534	948,514908	sp|P36897|TGFR1_HUMAN.HENILGFIAADNK.+2y9.light
721,37534	835,430844	sp|P36897|TGFR1_HUMAN.HENILGFIAADNK.+2y8.light
721,37534	631,340966	sp|P36897|TGFR1_HUMAN.HENILGFIAADNK.+2y6.light

721,37534	518,256902	sp|P36897|TGFR1_HUMAN.HENILGFIAADNK.+2y5.light
760,371417	814,478128	sp|P36897|TGFR1_HUMAN.NGTC[CAM]C[CAM]IADLGLAVR.+2y8.light
760,371417	743,441014	sp|P36897|TGFR1_HUMAN.NGTC[CAM]C[CAM]IADLGLAVR.+2y7.light
760,371417	628,414071	sp|P36897|TGFR1_HUMAN.NGTC[CAM]C[CAM]IADLGLAVR.+2y6.light
760,371417	515,330007	sp|P36897|TGFR1_HUMAN.NGTC[CAM]C[CAM]IADLGLAVR.+2y5.light

Table S5. Protein classification based on their biological functions.

**Transferases** (11%)	**Defense/ Immunity** *(21%)*	**Receptors** (26%)	**Signaling Molecules** (42%)
TGFR1_HUMAN	IL6R_HUMAN	TGFR1_HUMAN	IL6RA_HUMAN
TGFR2_HUMAN	IL2RG_HUMAN	IL10R1_HUMAN	TNFA_HUMAN
	IL10R1_HUMAN	IL10R2_HUMAN	FGF2_HUMAN
	IL10R2_HUMAN	IL1R1_HUMAN	PDGFA_HUMAN
		TGFR2_HUMAN	IL2RG_HUMAN
			IL8_HUMAN
			TGFB1_HUMAN
			VEGFA_HUMAN

Table S6. Validation test for CBUs and APB samples.

Tests	Peripheral Blood Units	Cord Blood Units	Acceptable Values
**Anti-HIV I/II**	Negative	Negative	<1.0 S/CO
**Anti-HBs**	Negative	Negative	<10 mIU/ml
**HBsAg**	Negative	Negative	<1.0 S/CO
**Anti-HBcore**	Negative	Negative	<1.0 S/CO
**Anti-HGV**	Negative	Negative	<1.0 S/CO
**Anti-HTLV I/II**	Negative	Negative	<1.0 S/CO
**Anti-CMV-IgG**	Negative	Negative	<1.0 AU/ml
**Anti-HCV**	Negative	Negative	<1.0 S/CO
**Anti-HAV**	Negative	Negative	<1.0 S/CO
**Anti-WNV-IgG**	Negative	Negative	<1.3 S/CO
**Anti-WNV-IgM**	Negative	Negative	<0.9 AU/ml
**Anti-T.Pallidum**	Negative	Negative	<1.0 AU/ml
**Anti-T.cruzi**	Negative	Negative	<1.0 AU/ml
**BacT/Alert aerobic**	Negative	Negative	-
**BacT/Alert anaerobic**	Negative	Negative	-
**Blood Agar**	No Growth	No Growth	<4-5 colonies
**Sabouraud Agar**	No Growth	No Growth	<1-3 colonies

Table S7. Validation test for PRP derived from CBUs and APB samples.

Tests	Peripheral Blood Units	Cord Blood Units	Acceptable Values
**Anti-HIV I/II**	Negative	Negative	<1.0 S/CO
**Anti-HBs**	Negative	Negative	<10 mIU/ml
**HBsAg**	Negative	Negative	<1.0 S/CO
**Anti-HBcore**	Negative	Negative	<1.0 S/CO
**Anti-HGV**	Negative	Negative	<1.0 S/CO
**Anti-HTLV I/II**	Negative	Negative	<1.0 S/CO
**Anti-CMV-IgG**	Negative	Negative	<1.0 AU/ml
**Anti-HCV**	Negative	Negative	<1.0 S/CO
**Anti-HAV**	Negative	Negative	<1.0 S/CO
**Anti-WNV-IgG**	Negative	Negative	<1.3 S/CO
**Anti-WNV-IgM**	Negative	Negative	<0.9 AU/ml
**Anti-T.Pallidum**	Negative	Negative	<1.0 AU/ml
**Anti-T.cruzi**	Negative	Negative	<1.0 AU/ml
**BacT/Alert aerobic**	Negative	Negative	-
**BacT/Alert anaerobic**	Negative	Negative	-
**Blood Agar**	No Growth	No Growth	<4-5 colonies
**Sabouraud Agar**	No Growth	No Growth	<1-3 colonies
**Endotoxin level**	Negative	Negative	<2.5 EU/ml
**Mycoplasma Test**	Negative	Negative	<0.72 MycoAlert Ratio (RLUs)
